# Taurine in health and diseases: consistent evidence from experimental and epidemiological studies

**DOI:** 10.1186/1423-0127-17-S1-S6

**Published:** 2010-08-24

**Authors:** Yukio Yamori, Takashi Taguchi, Atsumi Hamada, Kazuhiro Kunimasa, Hideki Mori, Mari Mori

**Affiliations:** 1 Institute for World Health Development, Mukogawa Women’s University, Nishinomiya 6638143, Japan

## Abstract

Taurine (T) was first noted as beneficial for stroke and cardiovascular diseases (CVD) prevention in genetic rat models, stroke-prone spontaneously hypertensive rats (SHRSP). The preventive mechanisms of T were ascribed to sympathetic modulation for reducing blood pressure (BP) and anti-inflammatory action. Recent epidemiological surveys revealed the involvement of inflammatory mediators in the pathogenesis of stroke and also atherosclerosis for which T was proven to be effective experimentally. Arterio-lipidosis prone rats, a substrain of SHRSP selectively bred for higher reactive hypercholesterolemia, quickly develop not only arterial fat deposition but also fatty liver which could be attenuated by dietary T supplementation. CARDIAC (CVD and Alimentary Comparison) Study was a WHO-coordinated multi-center epidemiological survey on diets and CVD risks and mortalities in 61 populations. Twenty-four-hour urinary (24U) T was inversely related significantly with coronary heart disease mortality. Higher 24U-T excreters had significantly lower body mass index, systolic and diastolic BP, total cholesterol (T-Cho), and atherogenic index (AI: T-Cho/high density lipoprotein-cholesterol) than lower T excreters. T effects on CVD risks were intensified in individuals whose 24U-T and -magnesium (M) excretions were higher. Furthermore, higher Na excreters with higher heart rate whose BP were significantly higher than those with lower heart rate were divided into two groups by the mean of 24U-T, high and low T excreters. Since the former showed significantly lower BP than the latter, T may beneficially affect salt-sensitive BP rise. Included among the typical 61 populations, were Guiyang, China or St. John’s, Newfoundland, Canada where in which the means of both 24U-T and -M were high or low, respectively. The former and the latter had low and high CVD risks, respectively. Australian Aboriginals living at the coastal area in Victoria were supposed to eat T- and M-rich bush and sea foods and be free from CVD 200 years ago, but they presently have nearly the highest CVD risks indicating that T- and/or M-containing seafood, vegetables, fruits, nuts, milk, etc, similar to prehistoric hunters’ and gatherers’ food should be good for CVD prevention. The preventive effects of T, good for health and longevity, first noted experimentally, were also proven epidemiologically in humans.

## Introduction

The health effects of taurine (T), “a wonder molecule”, was first noted in our genetic rat models for hypertension (Spontaneously Hypertensive Rats: SHR) [1,2] and stroke (Stroke-prone SHR: SHRSP) [3,4] and are now being confirmed epidemiologically by our WHO-coordinated Cardiovascular Diseases and Alimentary Comparison (CARDIAC) Study covering 61 populations in the world [5-8]. Advances in extensive studies on experimental models indicate that T is preventive against hypertension [9], stroke and atherosclerotic arterial diseases [10-12]. T is also expected to be effective for the prevention of nonalcoholic steato-hepatitis (NASH) increasing now in developed countries [13]. Accumulating evidence from CARDIAC Study indicates that common T intakes reduce cardiovascular disease (CVD) risks and contribute to the longevity of the Japanese [12] which have the lowest coronary heart disease (CHD) mortality in developed countries. Furthermore, the recent health study has revealed that CHD risks are definitely lower in the individuals and populations with higher 24-hour urinary (24U)-T and -magnesium (M) excretions [14].

### 1) Taurine (T) and prevention of cardiovascular diseases (CVD)

Since SHRSP was established as a genetic model developing stroke, extensive nutritional and pharmacological studies have been conducted experimentally [7,9,10,15]. Our experiments to feed SHRSP on high or low fish protein diets with or without 1 % salt in drinking water first demonstrated that fish protein rich in T attenuated salt-induced severe hypertension and decreased stroke incidence from 80% down to 10% [4,15]. High fish protein diet with low salt in drinking water was proven to be most effective to reduce stroke down to 0%. We further investigated the effects of several amino acids contained in fish protein in SHRSP and confirmed that T is effective for reducing blood pressure (BP) in SHRSP [9]. Among our experiments to prove the effect of various nutrient and amino acid supplementations on BP and stroke incidence in SHRSP, sulfur amino acids, such as T and methionine, were effective on the reduction of BP and stroke incidence [6]. Other than sulfur amino acids reducing BP, lysine had no effect on BP but was also effective for the prevention of stroke. We speculate therefore that enough amino acid supply to the vascular wall also might be protective against stroke [6,15].

Therefore, we further analyzed the nutritional mechanisms of stroke in SHRSP and also in humans. The common predilection site of stroke is the basal ganglia which are fed through perforating arteries branching recurrently from the cerebro-basal arteries. Blood flow through such recurrent arteries is reduced hemorheologically in hypertension, and thus vascular damage develops due to the reduced blood supply to these arterial walls [15,16]. Anatomically the vascular wall of these recurrent arterial branches should receive nutritional supply only from the blood stream inside the vessel through the blood-brain barrier, whereas the cerebro-basal arteries receive nutritional supply through capillaries around the vascular wall, (“vasa vasorum”). Our morphological electron microscopic studies in 1980’s for the first time demonstrated that vascular damages start at the outer layer of the vascular smooth muscle cells located at the furthest site from the vascular lumen [15,16]. Moreover, we proved that macrophages were activated in response to the vascular damages that occurred initially at the outer layer of perforating arteries in the brain. Such nutritional vascular damages are followed by inflammatory reactions. At the advanced stage vascular damages occur at the inner layer of cerebral vessels which leads to vascular wall rupture that causes cerebral hemorrhage or to thrombosis inside the damaged blood vessels that causes cerebral infarction [15,16]. These pathological processes were further confirmed in human autopsy materials by an immuno-histochemical technique [17] in which macrophages were recruited to cause and heal cerebro-vascular damages. Vascular smooth muscle cells initially remained intact near the intima close to the vascular lumen as in SHRSP. Therefore, we can speculate that the activation of macrophages and other inflammatory cells are supposed to further damage cerebral arteries, causing severe vascular lesions such as angio-necrosis. However, when these inflammatory cells contain much T from nutrients, T-chloramine neutralizes the adverse action of radicals produced from chlor [18] and may help vascular cells survive or regenerate to repair the wall [19].

After our demonstration of the importance of inflammation in stroke in rat models, the pathogenic concept of stroke as well as atherosclerosis in humans has also changed greatly [20]. High sensitive C-reactive protein (CRP), a marker and product induced by inflammatory reaction, was confirmed to be the reliable predictor of ischemic stroke and atherosclerotic CHD by extensive cohort studies [21,22]. Therefore, the control of local inflammation by T appears to be important for the prevention of stroke. The effect of fish protein on the incidence of stroke was experimentally first demounted in SHRSP in the 1970’s [4,15], and the cod protein feeding was reported to be clinically effective for reducing CRP in humans [23]. T or sulfur amino acids rich in fish protein may protect cerebral vessels through its anti-inflammatory action by the local formation of T-chloramines. Consistent with these studies, our world-wide epidemiological study showed that among 5 diet-related factors T/creatinine (Cr) ratio was proven to be a potent preventive factor against stroke as well as CHD as mentioned later [12].

### 2) Taurine (T) and lipotoxicity in the vascular wall and liver

By our further effort for establishing a better model of atherosclerosis from SHRSP, the selective sib-mating of the substrain of SHRSP, developing greater reactive hypercholesterolemia in response to high fat cholesterol (HFC) diet feeding, was started in 1972 [24]. The strain developed not only reactive hypercholesterolemia but also ring-like fat deposits in small mesenteric arteries within 2 or 3 weeks on HFC diet. This appeared to be a good model for studying the initial process of fat deposition on arterial walls in the development of atherosclerosis [25] because similar ring-like fat deposits were noted at autopsy in the atherosclerotic lesions of cerebro-basal arteries in the human brain.

Dietary T supplementation was proven to be effective for attenuating the development of hypercholesterolemia and for decreasing fat deposits in the mesenteric arteries of the selected SHRSP fed on HFC diets [26,27]. Such cholesterol-lowering effect was also confirmed in volunteer medical students who took 6g of T per day for 3 weeks [28]. These beneficial effects of T on hypercholesterolemia and arterial fat deposits were experimentally confirmed to be due to the increased bile acid production and the activation of 7α-hydroxylase [26], the rate limiting enzyme in the process of the conversion of cholesterol into bile acids. This has been proven to be due to the enhanced gene expression of cholesterol 7α-hydroxylase [29]. When T feeding lowered cholesterol levels, the gene expression was proportionally accelerated in relation to the reduced cholesterol level. Moreover, T supplementation was proven to effectively increase low density lipoprotein (LDL) receptors in the liver by an experiment of T supplementation accelerating the decay curve of radio-labeled (125I) LDL [30]. Therefore, cholesterol-lowering mechanisms of T are summarized to be due to an increase in LDL receptors and accelerated conversion into bile acids by T, and other mechanisms such as T’s effect to reduce intestinal acyl-CoA:cholesterol acyltransferase activity [26,31].

We are presently interested in the activation of LOX-1, a receptor for oxidized LDL that was up-regulated in SHRSP fed on HFC diet [32]. T actually decreased arterial fat deposition [26]. Therefore, T is speculated to attenuate the increase of LOX-1 receptors though a possible local antioxidant action by T-chloramine formation [19,31]. The suppressive effect of T on LOX-1 was noted actually in experimental diabetic or hypertensive models treated by T [33,34].

**Figure 1:**
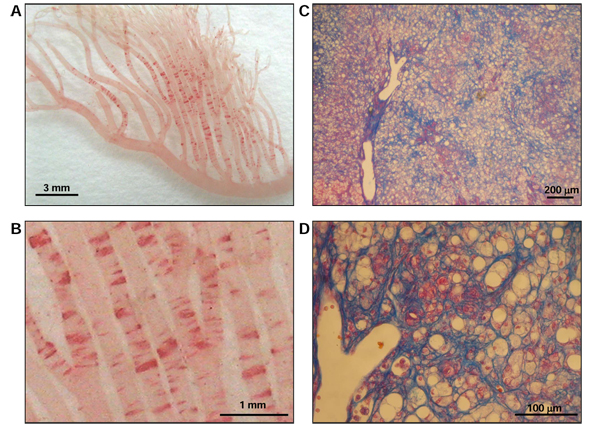
**Histopathological hallmarks of ALR fed a high fat and high cholesterol diet.** Ten-week-old ALR were fed a high fat and high cholesterol diet for 8 weeks. (A, B) Fat deposition in the mesenteric artery of ALR was detected by Oil red O staining and observed under low- and high-power fields. (C, D) Liver fibrosis of ALR was detected by Azan staining and observed under low- and high-power fields.

In relation to arterial lipid deposition we could establish a new model for NASH [35], now increasing in Japan as in other developed countries because of the westernization of dietary habit to eat more fatty meat and less fish. The substrain of SHRSP successively bred by checking greater reactive hypercholesterolemia and rapid arterial fat deposition for the last 37 years was named “Arterio-lipidosis prone rats” (ALR, SHRSP 5/Dmcr) [36]. The recent studies are confirming that ALR should also be a good model for NASH, developing not only fatty liver but also inflammatory reaction and active fibrosis forming pseudo-lobule as well as the typical morphology of Mallory’s hyaline bodies within 8 to 12 weeks of HFC diet feeding (Figure 1). Dietary T’s effect in this new model for NASH is expected, because subcutaneously injected T was reported to decrease serum lipid levels and serum transaminase levels, markers of hepatocyte damage as well as the biomarkers of oxidative stress in the rats fed on HFC diets developing fatty liver. Moreover, T suppressed hepatic mRNA expression of cytokines such as tumor necrosis factor-alpha and transforming growth factor-beta, possibly involved in the process of hepatocyte damage and fibrosis, whereas it increased the expression of a beneficial cytokine, adiponectin. However, the reported models are basically models for fatty liver and appear morphologically not to be typical models for NASH in humans as ALR. Since T supplementation was observed to decrease serum cholesterol levels and arterial fat deposition in ALR [36], if T could also improve ALR’s typical morphology of NASH, T would be “a wonderful molecule” for nutritionally protecting CVD risks and NASH, a common pathology in metabolic syndrome, and would therefore be expected to contribute to a healthy longevity in man.

In conclusion, our experimental studies consistently indicate greater prospective for T to contribute to the nutritional prevention of CHD, as well as stroke and probably NASH, the increasing health problem of mankind exposed to urbanization and westernization of dietary habit and life styles.

### 3) Taurine (T) and coronary heart diseases (CHD)

#### 3-1) T’s role in CVD prevention

These experimental findings to support the beneficial effect of T and other nutrients on CVD prevention led Y. Yamori to propose an international cooperative study on the association of dietary factors with CVD risks and CVD mortality in 1982. The protocol of this study obtained an international consensus of two meetings organized by WHO-Collaborating Center for Primary Prevention of CVD in 1983 and 1985 [5-7]. The CARDIAC Study is a multi-center cross sectional epidemiological survey for which about 100 males and 100 females aged 48 to 56 were randomly selected from each population and invited to participate in a health examination. This consisted of weight, height, BP measurement by an automated BP measurement system, blood tests for serum total cholesterol (T-Cho) and high density lipoprotein-cholesterol (HDL), as well as an analysis of biomarkers (the daily excretions and their ratios to Cr) of Na, potassium (K) M, T, isoflavones and urea nitrogen for estimating dietary intakes of salt, vegetables, milk products, seafood, soybean products and protein by sampling 24U [5-8,37,38]. The associations of nutrition with BP and with CVD mortalities were analyzed in “Core” Study and in “Complete” Study, respectively. “Core” Study demonstrated that the population averages of BP, both systolic and diastolic BP (SBP and DBP), were positively associated with the average population intake of daily Na estimated by 24U-Na excretion [7,37,38], and the population average of M/Cr in 24U were inversely associated with SBP and DBP indicating a significant association with DBP [7]. “Complete” Study showed significant positive association of the age-adjusted mortality rates of stroke with the population averages of 24U-Na excretion or 24U-Na/K ratio, and with the arachidonic acid ratio in the plasma phospholipids [8], but the rates were significantly inversely related with T-Cho. Moreover, CHD mortality rates were significantly positively related with the population averages of serum T-Cho and inversely related with polyunsaturated/saturated fatty acid ratios, n-3 fatty acid ratios of plasma phospholipids or 24U-T, indicating a protective effect of seafood intake on CHD [8,37].

**Figure 2:**
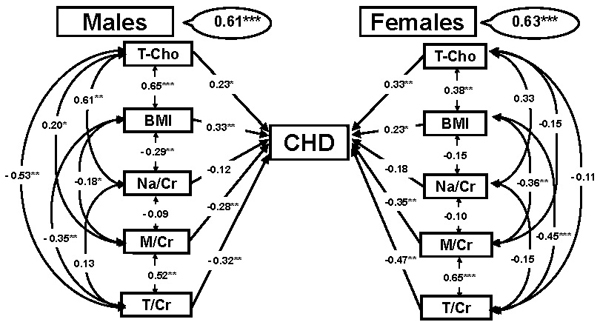
**CARDIAC Study results of structural equation modeling of coronary heart diseases (CHD).** Hypothetical pathway of 5 diet-related factors in relation to CHD. T-Cho: total cholesterol, BMI: body mass index (body weight (kg)/height (m)^2^), Na/Cr: 24-hour urinary (24U) sodium (Na, g) to creatinine (Cr, g) ratio, M/Cr = 24U-magnesium (M, mg) to Cr ratio, T/Cr = 24U-taurine (T, mmol) to Cr ratio. Significant difference: *p < 0.05, **p < 0.01, ***p < 0.001.

In CARDIAC Study populations we examined 5 diet-related markers in relation to CHD by applying the structural equation modeling for males and females. Five markers were T-Cho, BMI and Na/Cr, M/Cr and T/Cr ratios in 24U (Figure 2) [12,38]. The results clearly indicated that higher T/Cr ratios were highly significantly related to the risk reduction of CHD both in males and females, in contrast to T-Cho increasing the risk. Interestingly, our results first demonstrated M was also strongly related to the risk reduction following T. Since M content of seafood is also high, T and M are positively associated with each other. Twenty-four-hour U-T excretions were widely distributed in 61 populations’ averages, positively associated with seafood intakes and exceptionally high in all Japanese populations eating fish and seafood daily. With obviously less CHD mortality rates, Japanese data were excluded from the following analyses on the association of CVD risks with 24U-T or -M, and 3960 individuals from 41 WHO-CARDIAC Study populations were used for the following analyses [14].

#### 3-2) Influence of T, M and combined T and M on CVD risks

**Figure 3:**
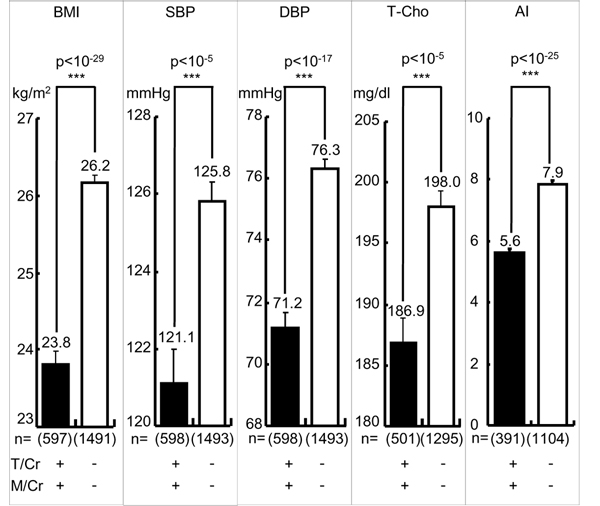
**Cardiovascular risks in higher taurine-magnesium (T-M) excreters and lower T-M excreters.** T/Cr = 24-hour urinary (24U) taurine (T, mmol) to creatinine (Cr, g) ratio, M/Cr = 24U-magnesium (M, mg) to Cr ratio. T/Cr: + ≧ mean (= 639.4), - < mean, M/Cr: + ≧ mean (= 82.8), - < mean. BMI: body mass index (body weight (kg)/height (m)^2^), SBP: systolic blood pressure, DBP: diastolic blood pressure, T-Cho: total cholesterol, AI: atherogenic index (T-Cho/high density lipoprotein cholesterol). Parenthesis indicates a number of participants. Significant difference: ***p < 0.001.

The mean of the individual data of 24U-T/Cr in 41 populations was 639.4. Individuals with 24U-T/Cr equal to or above and below the mean are defined as high T and low T eaters. High T eaters were proven to have significantly less CVD risks such as obesity (body mass index, BMI), both SBP and DBP, T-Cho and atherogenic index (AI: T-Cho/HDL), an indicator of the risk for developing atherosclerosis and CHD, than low T eaters [14]. Similarly, individuals with 24U-M/Cr equal to or above the mean 82.8 were proven to have significantly less CVD risks than those with 24U-M/Cr below the mean [14]. Therefore, we further analyzed these CVD risks in the individuals with both higher 24U-T/Cr and -M/Cr (≧ mean) (high T-M excreters) and proved that they had highly significantly lower BMI, SBP, DBP, TC and AI than low T-M excreters (< mean) as shown in Figure 3.

**Figure 4:**
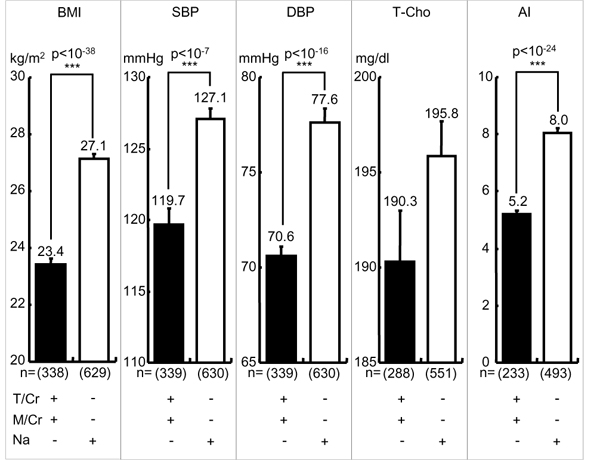
**Cardiovascular disease risks in higher and lower taurine-magnesium excreters with greater or lesser sodium excretion.** T/Cr = 24-hour urinary (24U) taurine (T, mmol) to creatinine (Cr, g) ratio, M/Cr = 24U-magnesium (M, mg) to Cr ratio, Na: 24U-sodium (Na as NaCl, g/day). T/Cr: + ≧ mean (= 639.4), - < mean, M/Cr: + ≧ mean (= 82.8), - < mean, Na: + ≧ mean (= 10.14), - < mean. BMI: body mass index (body weight (kg)/height (m)^2^), SBP: systolic blood pressure, DBP: diastolic blood pressure, T-Cho: total cholesterol, AI: atherogenic index (T-Cho/high density lipoprotein cholesterol). Parenthesis indicates a number of participants. Significant difference: ***p < 0.001.

**Figure 5:**
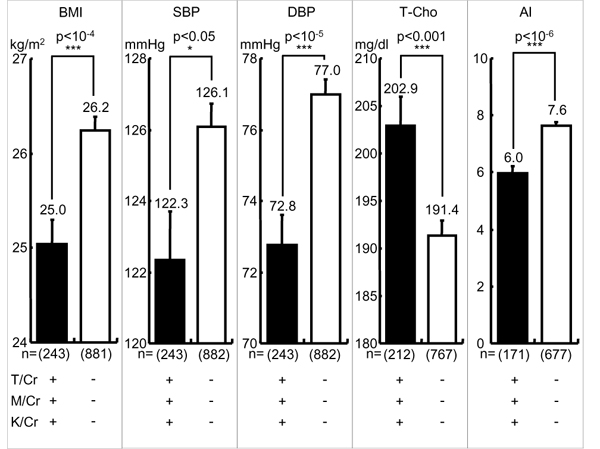
**Cardiovascular disease risks in higher taurine-magnesium-potassium (T-M-K) excreters and lower T-M-K excreters.** T/Cr: 24-hour urinary (24U) taurine (T, mmol) to creatinine (Cr, g) ratio, M/Cr: 24U-magnesium (M, mg) to Cr ratio, K/Cr: 24U-potassium (K, milliequivalent) to Cr ratio. T/Cr: + ≧ mean (= 639.4), - < mean, M/Cr: + ≧ mean (= 82.8), - < mean, K/Cr: + ≧ mean (= 46.1), - < mean. BMI: body mass index (body weight (kg)/height (m)^2^), SBP: systolic blood pressure, DBP: diastolic blood pressure, T-Cho: Total Cholesterol, AI: Atherogenic index (Total cholesterol/HDL cholesterol). Parenthesis indicates a number of participants. Significant difference: *p < 0.05, ***p < 0.001.

Since the differences in CVD risks between high T-M excreters and low excreters were statistically so high, we further analyzed the additional adverse effect of high salt (Na) intake on low T-M excreters or the additional beneficial effect of low Na intake on high T-M excreters (Figure 4). High and low daily T-M excreters were divided respectively into high and low excreters by the average of daily salt intake (10.2 g/day) estimated from 24U-Na. Differences in BMI, SBP, DBP and AI, except for T-Cho, remained statistically significant between high T-M with low Na excreters and low T-M with high Na excreters (Figure 4). In contrast to Na intake, K is regarded to beneficially affect CVD risks, particularly BP. Therefore, high and low T-M excreters were divided into high and low K excreters by the average of K/Cr ratio, 46.1 (Figure 5). Differences in CVD risks except for T-Cho remained statistically significant between high K, high T-M excreters and low K, low T-M excreters. High K and high T-M excreters showed significantly higher T-Cho than low K and low T-M excreters, indicating dietary habits to take more K linked to the diet to increase T-Cho higher. Therefore, we can conclude all these CVD risks (BMI, SBP, DBP, T-Cho, AI) are influenced more by differences in high and low T-M excretion than differences in Na or K excretion regarded generally as adverse or beneficial dietary factors.

### 4) Taurine and salt-related hypertension

**Figure 6:**
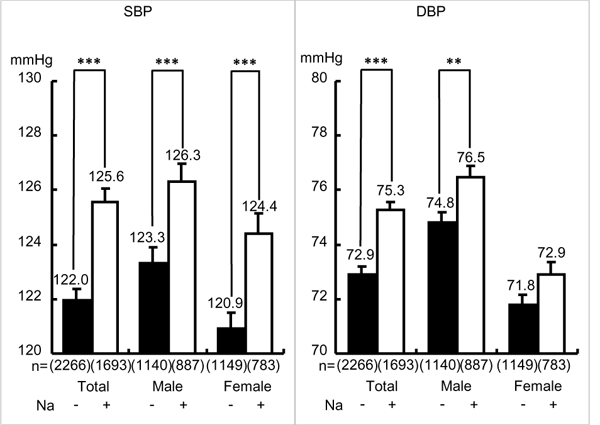
**Blood pressure in greater or lesser 24-hour urinary sodium.** Na: 24-hour urinary (24U) sodium (Na as NaCl, g/day), Na: + ≧ mean (= 10.14), - < mean. SBP: systolic blood pressure, DBP: diastolic blood pressure. Parenthesis indicates a number of participants. Significant difference: **p < 0.01, ***p < 0.001.

The population averages of both SBP and DBP in CARDIAC Study showed significantly positive association with the averages of 24U-Na excretion in males but not in females in general. The association was significant only in SBP of postmenopausal women, indicating gender difference in salt-sensitive BP rise [39]. Since gene-environmental interactions such as nutrition was supposed to be involved in the salt sensitively, individual participants in the CARDIAC health survey were divided into greater or lesser Na excreters by the mean (10.2 g) of daily salt intake estimated from 24U-Na excretion. As shown in Figure 6, greater Na excreters (≧ mean) showed significantly higher SBP in total and in males and females, respectively. However, in DBP the differences in total and males were significant, but there was no significant difference in females indicating the consistency of the individual data analyses with the population average data.

**Figure 7:**
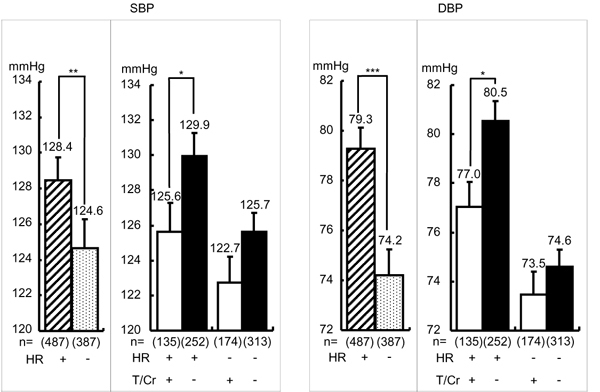
**Effect of taurine on blood pressure in male greater salt eaters with faster heart rate.** Male greater salt eaters were defined as shown in Figure 6 and analyzed only in those whose heart rate (HR) were recorded. T/Cr: 24-hour urinary taurine (T, mmol) to creatinine (Cr, g) ratio. T/Cr: + ≧ mean (= 639.4), - < mean, HR: + ≧ mean (= 72.9), - < mean. SBP: systolic blood pressure, DBP: diastolic blood pressure. Parenthesis indicates a number of participants. Significant difference: *p < 0.05, **p < 0.01, ***p < 0.001.

Heart rate (HR) is related to CVD risk in CARDIAC data [40] as well as in other epidemiological data indicating lower HR is significantly related to longer life-expectancy in the elderly [41,42]. Since CARDIAC Study analysis on the population averages showed that higher 24U-T was significantly associated with lower BMI, SBP, DBP and HR, the involvement of T in salt-sensitive BP rise was analyzed by dividing greater Na excreters in males into those with higher HR (≧ mean, 71.5 beat/min) and with slower HR (< mean). As shown in Figure 7, greater Na excreters with higher HR had significantly higher SBP and DBP than those with lower HR, indicating higher HR with higher salt intake estimated by greater 24U-Na excretion was related with salt-sensitive BP rise in which sympathetic neural component was supposedly involved. Therefore, greater 24U-Na excreters with higher and lower HR were both divided into high and low T excreters (T/Cr ≧ or < mean). Higher T excreters’ SBP and DBP were significantly lower only in greater Na excreters with high HR, and there was no significant difference in both SBP and DBP of greater Na excreters with lower HR. These findings are suggestive of T’s BP reducing effect on neural regulation of BP rise induced by salt loading as in experimental studies in rat models of salt-sensitive hypertension, SHRSP and DOCA salt hypertensive rats. T administration decreased BP more markedly in salt-sensitive SHRSP than in SHR and in normotensive Wister-Kyoto rats [9], and in salt-loaded DOCA hypertensive rats in which T reduced BP increase as well as catecholamine excretion [43]. The latter indicates the neurogenic development of salt-induced hypertension. We previously reported that BP and catecholamine responses to cold exposure stress in young volunteers were significantly greater in those with family history of hypertension than the other without the family history [15,44]. Therefore, our present epidemiological data indicating that among greater Na excreters with greater HR, higher T excreters showed lower BP than lower T excreters support the possible neurogenic component modulated by T in the salt sensitive hypertension.

### 5) Taurine (T) and magnesium (M) in high and low risk populations

**Figure 8:**
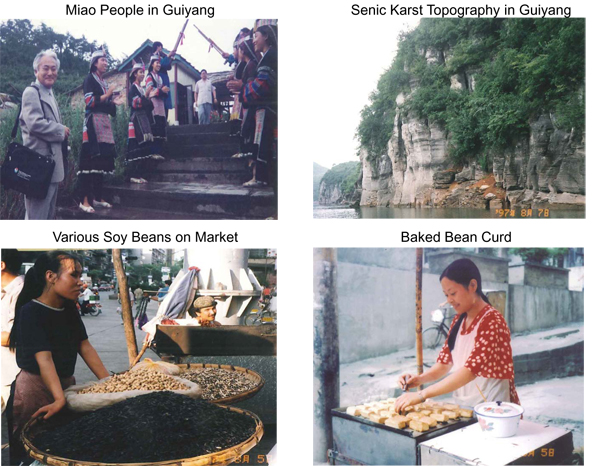
Food culture, lifestyle and topographic background of Guiyang people in China.

**Figure 9:**
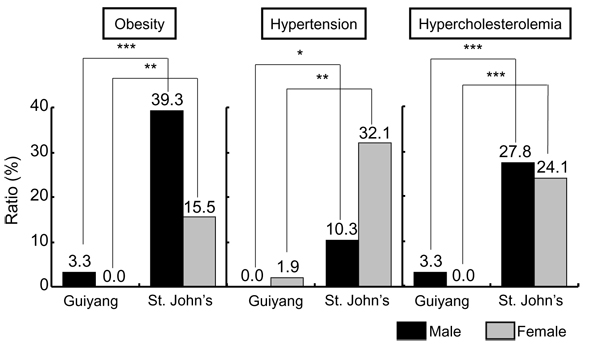
**Prevalence of risk of lifestyle-related diseases in populations excreting higher and lower taurine-magnesium.** Obesity: BMI (body mass index, (kg)/height (m)^2^) ≧ 30, Hypertension: systolic blood pressure ≧ 140 mmHg and/or diastolic blood pressure ≧ 90 mmHg, Hypercholesterolemia: total cholesterol ≧ 220mg/dl. Significant difference: *p < 0.05, **p < 0.01, ***p < 0.001.

Since the individuals with greater T and M intakes estimated from 24U excretions had significantly lower CVD risks than lesser T and M excreters [14], typical populations, in which the population averages of both 24U-T and -M were higher and lower than the average of CARDIAC population samples, were investigated for their life style and dietary habits for comparison. Among 41 WHO-CARDIAC populations, Guiyang people in south-west China near Guangzhou showed both higher 24U-T and -M than the averages among total CARDIAC populations (Figure 8) [38]. In contrast, St. John’s population living on Newfoundland Island, Canada had both lower T and M than the averages of the CARDIAC populations. In Guiyang we first carried out a health survey in 1988 and repeated the survey in 1997 after the rapid economic development in China [38]. There lived Miao, Pui and other minority people who were still keeping a traditional dietary habit, such as eating daily soy beans and various soy products, baked bean curd (coagulated soy protein) prepared by M-rich water and eating much fresh water fish, indicating that their rich sources of T and M are fresh water fish, soy and soy products. The prevalence of CVD risks was remarkably low in Guiyang, and they had almost no risks of obesity, hypertension and hypercholesterolemia indicating that their dietary habit of consuming high levels of both T and M is ideal for CVD prevention (Figure 9). Correspondingly, the Guiyang population had significantly lower BMI, both SBP and DBP, T-Cho level and AI than the averages of CARDIAC populations taking various amounts of T and M in other parts of the world. In contrast, Newfoundlanders in St. John’s were the descendants of Scottish and Irish immigrants and were engaged in farming sheep on the island and also in fishing for abundant sea water fish near the island. They traditionally salted fresh fish in barrels for shipping them as preserved food to Canada and US, and did not eat raw fish like the Japanese. They were eating mainly meat products so that their T and M excretions were lower than the averages of CARDIAC populations. CVD risks of Newfoundlanders both in male and females were significantly higher than those of the Guiyang people who were consuming high levels of both T and M (Figure 9). St. John’s population was known for their high CVD mortality rates, whereas Guiyang’s population was known for their low CVD mortality rates and their longevity in China. These data based on the individual biomarkers related to T and M intakes indicate that eating foods containing both T and M is good for the physiological regulation of the homeostasis of the cells and human bodies. This dietary habit may contribute to the prevention of CVD and thus to the healthy longevity of humans. CVD risks are definitely lower in individuals excreting high levels of 24U-T and -M. Among CARDIAC populations, Guiyang population excreting and supposedly taking both had definitely less CVD risks than the St. John’s population who were not excreting and taking both elements. Therefore, seafood, soy, nuts, and milk taken regularly as the sources of T and M could be recommended for CVD prevention.

### 6) Bush food rich in T and M, needed for health

**Figure 10:**
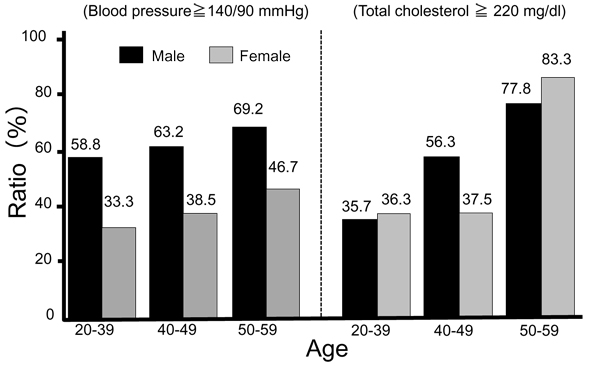
**Prevalence of hypertension and hypercholesterolemia.** Hypertension: systolic blood pressure ≧ 140 mmHg and/or diastolic blood pressure ≧ 90 mmHg, hypercholesterolemia: total cholesterol ≧ 220mg/dl.

**Figure 11:**
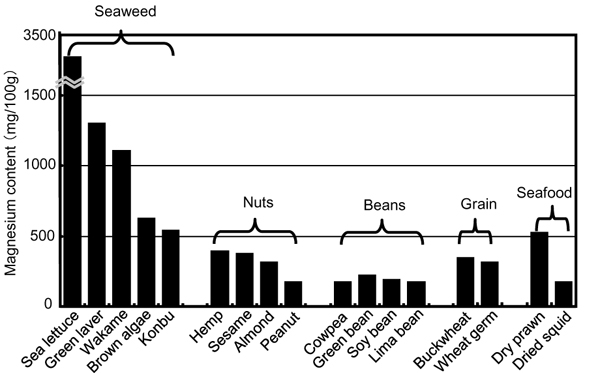
Magnesium contents of typical dry food.

**Figure 12:**
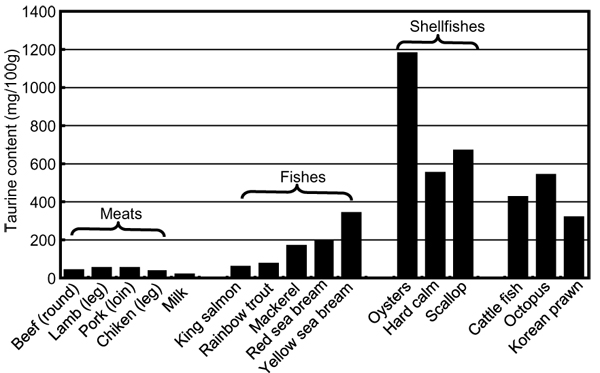
Taurine contents of typical food.

We are still continuing our CARDIAC health examination since we started the world wide study in 1985 in coordination with WHO. Our recent study on Australian Aboriginal people for over past 7 years demonstrated that they are presently suffering most severely from lifestyle-related diseases in comparison with the other CARDIAC populations [38,45]. Obesity, hypertension and diabetes in Aboriginals start 20 to 30 years younger than other Australian and Japanese people (Figure 10) [45]. When they lost bush food, their M intake was greatly decreased because bush food consisting of nuts, seeds, grains, beans, fruits and seaweeds contains much M (Figure 11). Aboriginals living near the coast of the state of Victoria used to utilize various foods from the sea and fresh water. Aboriginals living in Framlingham, Victoria were known to start the culture of eels 8,000 years ago and there were several large shell mounds, the piling up of various shells like mountains at the sea side not far from Melbourne, indicating that Aboriginals’ life had been highly dependent on their bush food as well as on the seafood, both the richest sources of T and M. Before the colonials were established, their daily foods were clams, various shells and fish containing a large amount of T (Figure 12), and also their traditional smoked eels, which were a rich source of T and DHA and eaten as their common preserved food. As hunters and food gatherers, Aboriginals, according to the 2-week food intake analysis report, were supposed to live by taking their energy, for example, 34% from complex carbohydrates, 13% from fat and 53% from protein [46]. Although we do not know exactly what they ate, we tentatively suppose, for example, they ate 400 g of yam, 300 g of frogs, 5 g of almonds, 140 g of bream, 200 g of clams, 300 g of snails. These foods contain about 100 g of carbohydrates, 15 g of fat and 170 g of protein and correspond roughly to 1300 kcal/day. The daily intake of T and M from these foods is about 3200 mg and 640 mg, respectively, both being far higher than the average of CARDIAC populations in the world.

Moreover, they had no salt at all before Captain Cock reached Australia at the beginning of 18th century. Our CARDIAC data help us to speculate how Aboriginals should have been free from CVD risks, based on our comparison of CARDIAC Study individuals consuming a high amount of T and M but less Na with those consuming less T and M but a great amount of Na. CVD risks were clearly and highly significantly lower in the CARDIAC Study participants taking a high amount of T and M but less Na, corresponding to the nutritional balance of Aboriginal ancestors eating traditional bush food. High T and M but less Na-containing foods were supposed to be taken commonly by the ancestors of humans living on hunter-gatherers’ foods like Aboriginal ancestors for nearly 10 times longer than the period after the start of agriculture and farming. Therefore, these dietary conditions seem to be more suitable for the human genome to live a healthy life without CVD.

### 7) T and M in cardiovascular pathophysiology and longevity

Both T and M are basically important for the maintenance of life. T is involved in cellular physiology by its effect on osmoregulation, anti-oxidant, membrane stabilization and calcium regulation [47-49], and also on lipid metabolism related to dyslipidemia and atherosclerosis through its role in bile acid conjugation [47,48]. Recently, T-conjugated endogenous bile acid derivative, ursodeoxycholic acid was focused on as a chemical chaperone which was proven to reduce endoplasmic reticulum (ER) stress and restore glucose homeostasis in a mouse model of type 2 diabetes [50]. The alleviation of ER-stress may restore insulin sensitivity in the liver, muscle and adipose tissue, thus contributing to the resolution of fatty liver diseases, diabetes and obesity. High dietary T administration was proven to reduce apoptosis and atherosclerosis possibly via normalization of ER stress [51].

Moreover, in relation to the ageing of vascular tissue and the accelerated senescence of endothelial progenitor cell (EPC) noted in SHRSP [52], T was reported to attenuate EPC senescence in SHRSP and to modulate clinically the deterioration of endothelial function in smokers [53-55]. These classical and new concepts of the pathophysiological roles of T suggest that a high T intake may contribute to longevity through CVD health and lifestyle-related diseases.

On the other hand, M is the 8th abundant element in the weight, the number of atoms and the volume percentage of all atoms on the earth. It is also the most abundant intracellular divalent cation and is involved in the various biological functions of about 300 enzymes as their coenzyme. They include all enzymatic reactions requiring ATP, such as Na-K ATPase, important for intracellular ionic balance. Therefore, M is supposed to be causatively and clinically related to cardiovascular health, hypertension and diabetes [56-58]. Lowering of intracellular free M was observed in SHRSP in the process of aging and development of hypertension [59]. Dietary M supplementation increased intralymphocytic free M and attenuated the grade of hypertension [60]. These experimental findings were recently confirmed clinically in patients with mild hypertension whose ambulatory BP was significantly decreased concomitantly with the increased intracellular free M and K, and decreased intracellular free Ca and Na, by the dietary supplementation of 600 mg of pidolate M [61]. In this clinical experiment, M supplementation increased serum M and 24U excretion of M. Therefore, the aforementioned epidemiologically observed 24U-M excretion was regarded to correspond to dietary M intake. M was also reported to decrease BP by inhibiting sympathetic nerve by blocking N-type Ca channels [62].

Since the evolutional origin of life of human beings was inside the sea containing abundant M and food gatherers lived on seafood rich in T, both M and T are assumed to be essential for the homeostasis maintaining cardiovascular health.

## Conclusions

In addition to the pathophysiological and various physiological functions of T, the preventive effect of T was first experimentally demonstrated in hypertensive rat models, SHR and SHRSP developing hypertension and stroke genetically. World-wide epidemiological studies conducted for the last 25 years revealed that 24U-T was inversely associated with the age-adjusted mortality rates of stroke and CHD. High 24U-T, particularly combined with high M excretion, was associated highly significantly with lower CVD risks, obesity, hypertension, hypercholesterolemia and AI. These findings indicate the consistency of the beneficial T effects on animal models and humans proven either experimentally or epidemiologically. Such consistency of the extensive basic and epidemiological findings of T effects and the effects combined with M indicates greater prospective for T to contribute to the nutritional prevention of CVD and lifestyle-related diseases.

## List of abbreviations used

AI: atherogenic index, BMI: body mass index, ALR: arterio-lipidosis prone rats, Ca: calcium, CARDIAC Study: Cardiovascular Diseases and Alimentary Comparison study, CHD: coronary heart diseases, Cr: creatinine, CRP: C-reactive protein, CVD: cardiovascular diseases, DBP: diastolic blood pressure, ER: endoplasmic reticulum, HDL: high density lipoprotein, HR: heart rate, K: potassium, LDL: low density lipoprotein, M: magnesium, Na: sodium, NASH: nonalcoholic steato-hepatitis, SBP: systolic blood pressure, SHR: spontaneously hypertension rats, SHRSP: stroke-prone spontaneously hypertension rats, T: taurine, T-Cho: total cholesterol, 24U: 24-hour urine.

## Competing interests

The authors declare that they have no competing interests.

## Authors’ contributions

YY designed taurine-related studies and prepared for this review. MM and HM conducted epidemiological surveys. TT contributed to the data analysis. AM conducted laboratory analyses. KK helped experimental studies and reference collection.
